# British South Asian and Muslim Community’s Perceptions and Experiences of Addiction and Traditional Drug and Alcohol Treatment Services

**DOI:** 10.3390/ijerph21101338

**Published:** 2024-10-10

**Authors:** Zeibeda Sattar, Lydia Shrimpton, Hayley Alderson, Monique Lhussier, Ruth McGovern, William McGovern

**Affiliations:** 1 Department of Education and Community Wellbeing, Faculty of Health and Life Sciences, Social Work, Northumbria University, Coach Lane, Newcastle upon Tyne NE7 7XA, UK; lydia.lochhead@northumbria.ac.uk (L.S.); monique.lhussier@northumbria.ac.uk (M.L.); william.mcgovern@northumbria.ac.uk (W.M.); 2 Faculty of Medical Sciences, Population Health Sciences Institute, Newcastle University, Richardson Road, Newcastle upon Tyne NE2 4AX, UK; hayley.alderson@newcastle.ac.uk (H.A.); r.mcgovern@newcastle.ac.uk (R.M.)

**Keywords:** cultural competency, South Asian, Muslim, stigma, barriers, drug and alcohol, treatment services, recovery, substance use, accessibility

## Abstract

Within traditional drug and alcohol (D&A) treatment services, the majority of those accessing support are of white ethnicity, with only a small percentage of people from the British South Asian (BSA) and Muslim community engaging in treatment services. This paper aims to explore perceived barriers to accessing traditional D&A services within the British South Asian and Muslim communities, based on qualitative data from interviews with family members and a practitioner. Qualitative data were obtained via 11 semi-structured interviews involving a practitioner (n = 1), and family and friends (n = 10) of those with historic and current D&A use in the community. Reflexive thematic analysis revealed four themes: (1) awareness of drug and alcohol use in the community, (2) drug and alcohol use as a taboo topic and the impact of admitting use, (3) knowledge of services for treatment, (4) how to increase awareness and accessibility of treatment. There was an increasing awareness of D&A use in the BSA and Muslim community. Despite this, limited conversations and misconceptions around D&A use and recovery led to those using D&A and their family members feeling stigmatised within their community and unable to seek support. This paper concludes by recommending increased communication between the BSA and Muslim communities and D&A treatment services to ensure accessibility of treatment by improving cultural competency.

## 1. Introduction

Across the UK, there has been an increase in drug and alcohol (D&A) use across all communities, including minority ethnic groups (Office for National Statistics) [1]. As a note, this study refers to both drug and alcohol addiction, only mentioning drugs or alcohol if reference is made to one or the other specifically. Despite this increase in D&A use, traditional D&A treatment services continue to be represented by predominantly white groups, with minority ethnic groups, such as British South Asians (BSAs) remaining under-represented. The BSA community is defined as people who live in the UK and descend from Pakistan, India and Bangladesh, with this ethnic group and the Muslim religion group being the focus of this study. Within the BSA and Muslim community, D&A use is prohibited and viewed as a sin [2]. Despite this, there has reportedly been a dramatic and concerning rise in D&A use observed in the younger second and third generation of BSAs and Muslims globally [3,4,5]. This younger generation of BSAs are reportedly shunning their traditional culture and values, with an assumed growing influence of Western society, particularly in terms of exposure to liberal values and greater social autonomy, as an impact of deprivation [6].

Qualitative data from the perspectives of those who use drugs and alcohol and those who support them is limited regarding the BSA and Muslim community [4,7,8], which is partly linked to the widely held misconceptions that they do not use drugs and alcohol [3]. This has led to an acknowledged lack of socially and culturally sensitive services, interventions and treatment options and a lack of satisfaction in people’s experiences when they do access services [2,5,9]. Only 1% of those accessing D&A treatment in the UK are BSA, which is not reflective of the proportion of BSA members actively using drugs [7,8].

There is an emerging field of research which has so far focused on the prevalence of D&A addiction in South Asian communities in the UK. Given that it is known that there is a high morbidity and risk of D&A issues amongst the BSA and Muslim communities, narratives are heterogeneous and lack specific evidence in the narrative to unpick the complex intersections among the community and D&A services regarding accessibility. This qualitative paper uniquely adds the perceptions and lived experiences of the South Asian community and their experiences with D&A services. More work, however, is required to explore the perceptions, recovery needs and experiences of the BSA community regarding D&A use, and their engagement and experiences with treatment services.

In 2020, it was estimated that 284 million people globally aged between 15 and 64 years old had engaged in drug use at least once [10], with 2.6% (862,000) of adults in the UK reporting that they engage in drug use on a regular basis [1]. Latest figures show population increases in the SA community [11] from 7.5% to 9.3% between the last two censuses, a respective breakdown being: Pakistani 2.7%; Indian 3.1%; and Bangladeshi 1.1%. This increase is also reflected in the study area, a northern city of England, in which the SA community increased to 11.4% from 9.7%. Similarly, the 2021 Census revealed a local increase in the population of the northern city of England concerning residents identifying as Muslim, with 9% (26,896) of residents responding as belonging to the Muslim religion, in comparison to 6.7% the year prior [12].

With regard to D&A use in the BSA community, the latest government survey reported 3.4% of Asian adults in England to have used illicit drugs 12 months prior to the 2014 survey, with mainly males recorded as engaging in drug use, compared to females [13]. Given the fact that these data are self-reported and the likely religious and cultural barriers to reporting, this figure is likely to be an underestimation. In parallel, academics have highlighted the fact that BSA communities are less likely to access drug and alcohol treatment [14,15,16]. Not all members of the BSA community are Muslim and share Muslim standpoints; however, Islam has been acknowledged as influential in shaping South Asian culture [17]. The Islamic stance is that alcohol and drugs cause intoxication and are thus prohibited [18,19]. However, D&A use is still prevalent amongst the Muslim community [14,19], with an increased normalisation of cannabis use observed amongst BSA youth in the UK [20]. This research coincides with the latest government survey that suggests there may have been an increase in illicit drug use from 2014 to 2021 amongst BSA adults in England.

Barriers to treatment are common for people from minority ethnic groups [21]. It has long been acknowledged that minority ethnic peoples are less likely to access treatment [22]. When it is accessed, it is often due to a related health issue [23], with this lack of direct access being linked to cultural norms [16]. Although cultural norms and values have often been a protective factor in prohibiting BSA community members from using drugs in the first place [21], it has also led the BSA and Muslim community to hide their D&A use [16]. This concealing of D&A use has been linked to shame and a fear of exclusion from the Islamic community, families and some imams [2,16]. This has been perpetuated by the community’s portrayal of those who use drugs being “sinners” and unintentional overdoses being equated to suicide—a prohibited act in Islam [24]. Drug use has been considered a “taboo” topic, meaning those who use drugs are unable to openly discuss their drug use with their community [25,26]. This discretion has been perpetuated by the reported collective denial amongst the Muslim community that drug use is prevalent in the community. This denial has also been found amongst community leaders, despite their power to provide community-based resources related to addiction and recovery, resulting in drug use being unrecognised by community members [26,27].

*Barriers to Treatment and Stigma*

Historically, there have always been negative stereotypical beliefs about people who use drugs (PWUD) [28], with this labelling and stigma creating a barrier for PWUD to access traditional D&A treatment services for fear of further judgement and discrimination [29]. A recent systematic review reported several misconceptions amongst the South Asian community regarding how traditional D&A treatment services can be accessed and what this involves, with false information creating a barrier to seeking recovery support [14]. These included the misconception that bribery or payment is needed to access harm reduction services [30]; that treatment is ineffective; that it is not confidential [25,31,32]; and that D&A services are generally not trustworthy [21,33]. It has also been reported that there is a lack of education on what addiction actually is amongst the Muslim community [34]. Another barrier to treatment for the Muslim community is that members believe a non-Muslim practitioner will not be able to provide a service reflective of and including key aspects central to the Muslim lifestyle, such as social, spiritual and historical outlooks. For example, some members of the South Asian and Muslim community (specifically Bangladeshi and Pakistani) are forbidden to drink from a religious perspective, and drinking is not part of their culture when compared to wider society [21,25,35]. This, in turn, results in concerns regarding cultural competency, and that treatment is currently white-orientated and unable to be responsive to and accommodate the needs of such minoritised communities [2,9].

Stigma has also been recognised as a barrier for the South Asian community to access drug and alcohol treatment [21,25,35]. Stigma involves an individual being devalued based on their attributes, characteristics and/or behaviours, with this being inclusive of religion and often leading to active discrimination [8,36]. Stigma has been linked to preventing people from accessing treatment due to the individual perceiving that they will be stigmatised in this setting, with PWUD often being stigmatised and labelled negatively in society [24,37]. Psychological factors, such as emotional adjustment and responses to crisis situations, have been shown to significantly influence individuals’ willingness to seek help and access treatment services [38]. In terms of the South Asian community, stigma has played a role in drug and alcohol use, in that negative attitudes towards PWUD have led to families then stigmatising the individual and excluding them from their own family network and community [30,37,39]. This has obstructed recovery for individuals as they avoid accessing D&A services due to believing that if they do so, their usage will no longer be concealed and will negatively impact their family’s reputation [40]. This has further encouraged the belief amongst the Muslim community that addiction is a private and shameful matter not to be shared outside of the family network [41]. In particular, women in the South Asian community have been noted as under-represented in D&A treatment services [16], with BSA women in Birmingham and London (locations with large Muslim populations) reporting that they are unaware of services tailored to them in their communities [42]. It has also been suggested that the reluctance of BSA women to seek help for their alcohol and/or drug use is due to the fact that drug use does not adhere to the BSA women’s traditional role [31]. This is in light of research looking at American Muslim university students where there was no evident gender disparity regarding drug use [42]. However, despite there being no difference in the frequency in which women and men use drugs and alcohol in the community, recently, it was highlighted that there is a gender disparity, culturally, in terms of tolerance of addiction issues, with women being found to be judged harsher for alcohol use compared to men [41].

## 2. Materials and Methods

### 2.1. Study Design

This study was a qualitative project that utilised semi-structured interviews conducted between November 2023 and February 2024. Qualitative research employs a constructivist philosophical position and attempts to explore in rich detail the phenomenon of interest. This involves the researcher becoming immersed in the data and attempting to gain a holistic, rather than reductionist, understanding of the phenomenon or experience and was therefore most appropriate for this study [43,44,45].

### 2.2. Participants

The researchers (ZS and LL) conducted the fieldwork by recruiting participants from the BSA and Muslim community by approaching local D&A treatment services. The project team also recruited participants from the community who were known to be caring for someone in the community who was in recovery and/or engaging in D&A use and identified by local community ambassadors. This recruitment was possible because a member of the research team (ZS) grew up within this local BSA and Muslim community and had prior access and knowledge of some of the local D&A issues. ZS is a well-respected member of the community, and participants were open to discussing this topic, although some were still very reserved and apprehensive about sharing the experiences they had. The inclusion criteria were as follows: BSA adults, aged over 18 years old, who lived in the north-east, and had experience with D&A use or were a family member/friend of BSA community members using drugs and alcohol.

Potential participants were approached by the research team with information about the project and asked if they would like to or know someone who they believe would like to take part in a qualitative interview. From this, one practitioner working in a drug and alcohol service and ten members of the SA community were recruited to take part in a qualitative semi-structured interview (n = 11). Eight BSA participants had family members or friends) who engaged in alcohol and/or drug use; four with family/friends currently using D&A; and four with family/friends who historically used D&A. Two participants were community leaders/ambassadors in the SA community.

Nine participants were female, and one participant was male. BSA participants were aged between 28 and 55 years old (mean age: 41.36), and all identified as Muslim. Their ethnicities were Bengali; Pakistani; and Indian. Interviews took part either in person or via telephone (6 in person; 5 telephone interviews), in accordance with the preference of the participant based on availability and comfort. It is noted that ZS (PI) was regarded as a respected member of the community and the participants were familiar with her as a member of the community. This was reflected in the fact that participants were happy to discuss the topic over the telephone without hesitation if they were unable to do face-to-face interviews. Also, there were more female participants, which may have been because ZS was also female and had stronger links with women in the community than the male members. It was felt that more information could have been obtained with face-to-face interviews; however, the standpoint that telephone interviews were better than no interviews was taken. All interviews lasted between 30 and 60 min.

Table A1 here (please see Appendix A).

To recruit participants, efforts were also made with the local D&A services including a carer forum for the BSA community. ZS (PI) and LL have extensive contacts within the city and among these groups. Due to the nature and sensitivity of this project, an opportunistic approach was conducted to draw on a broad range of experiences and insights. The research design allowed us to develop a deeper and more comprehensive understanding of D&A use within the BSA community, and the barriers perceived by the participants. The participants required reassuring many times prior to the interviews; hence, it took a lot longer than initially predicted for the field work. ZS attended several community events and religious gatherings to ensure a rapport was built up with participants, although she had networked with them at different levels in the community.

### 2.3. Analysis

The approach taken for this study was inductive. Inductive methods are applied to qualitative research design and theory that are developed from the ground up, rather than deductive methods [46]. As the research aims of this study are exploratory and seek to understand BSAs’ and Muslims’ experiences of D&A treatment and use, this study design is informed by an interpretivist paradigm [47]. By doing this, the research team were equipped to position the research to be able to construct knowledge through lived experiences and interactions with other members of society and research to ensure that the knowledge produced is reflective of their reality [45].

Reflexive and inductive thematic analysis was undertaken following the steps outlined by Braun and Clarke (2006, 2019) [48,49] who argue that thematic analysis offers the researcher the ability to provide a comprehensive, descriptive and detailed account of their thought processes, feelings, meaning constructs and mental maps. It does not only identify and analyse patterns [50] but also interprets hidden and subtle aspects of the concerned phenomena [50]. ZS was also able to pick up subtle turns of phrases and body language because she was from the SA community and was bilingual (Urdu), which enabled deeper emersion in conversation and data.

The interviews were digitally recorded (with consent) and transcribed verbatim. Identifiable information was removed at transcription. All data collected in this study were anonymised and kept in accordance with Northumbria University procedures. NVivo was used to facilitate the analysis of the data. After reading a few transcripts, the initial coding strategy was identified/emergent and then categorised into broader topic areas/themes. To enhance data analysis rigour, sample transcripts were circulated to team members and discussed, and codes agreed.

Analysis identified four themes, presented in turn below: (1) awareness of drug and alcohol use in the community, (2) drug and alcohol use as a taboo topic and the impact of admitting use, (3) knowledge of services to treatment, (4) how to increase awareness and accessibility of treatment.

### 2.4. Ethical Considerations

This research project was approved by the Ethics Committee at Northumbria University (Project Reference Number: 4770).

## 3. Results

The majority of those accessing support in traditional drug and alcohol (D&A) treatment services are of white ethnicity, and only a small percentage of people from the British South Asian (BSA) and Muslim communities engage with treatment services. This study has explored perceived barriers to accessing traditional D&A services within the BSA and Muslim communities.

### 3.1. Awareness of Drug and Alcohol Use in the Community

This theme discusses how the BSA community acknowledges their awareness of drug and alcohol use amongst their community, and what the nature of the addiction is in terms of drugs and alcohol according to what generation a person belongs to.

All participants reported that they were aware of D&A use and that drugs (particularly cannabis and cocaine) and alcohol were prevalent in their community. Despite this awareness, those in the BSA and Muslim community were perceived as refusing to acknowledge the issue and talk about addiction with their peers:

*“There’s drug and alcohol issues within the Asian community hugely, but because it’s not discussed about, or it’s sort of hidden, it’s a really taboo topic.”*

P3—daughter of a person who used drugs

It was highlighted by a couple of participants that this shielding of addiction in the community was also carried out by those close to the person consuming and supplying drugs:

*“When it comes to dealing drugs, I feel like… The families and friends do know, but they don’t speak of it.”*

P8—family member of a person who used drugs

Participants described how they believed awareness of D&A was increasing within the community. It was stated that there was a recognition now that it is prevalent in the BSA community, especially amongst the younger generations:

*“I think now, talking-wise, it’s improving. I think it’s more like… People are talking about it now. You know? Because it’s everywhere. Drugs and alcohol are everywhere.”*

P1—carer of a person who used drugs

As seen in the above quote, it was suggested that conversations are increasing as D&A use increases in the BSA community, with the topic becoming less avoidable. It was also noted by a participant that it has become more difficult to ignore drug and alcohol use now that they are being used more openly in the community:

*“Living in the area […] you do see it openly as well, like, drugs being dealt, people taking drugs, alcohol being drunk on the street and things like that.”*

P9—family member of a person currently using drugs

It was noted that the younger generation were perceived as more willing to discuss drug and alcohol use, and to engage in D&A use themselves. It was highlighted by over half of the participants that not only is D&A use increasing, affecting the rate at which conversations around the topic are taking place, but also that they believe there is a generational difference in the perspective of D&A use and treatment, as well as mental health, with social media often utilised as a platform for these topics:

*“In today’s day and age, you’ve got a lot more young people using, with like, young lads and young girls, so it’s more talked about now and obviously you’ve got a lot of people in and out of prison and it’s become more and more […] you’ve got a lot of young men and women that are openly talking.”*

P3—daughter of a person who used drugs

D&A use amongst the younger generation was spoken about negatively by a community member, with them feeling this was a reflection on how much the individual cared for their community. This was stated in contrast to the older generation, although it was noted that despite the younger generation’s perceived openness to use drugs and alcohol, there was still a level of discretion that they took when it came to consuming substances:

*“The British Pakistani community, the Asian culture, I feel like it’s very generational, so the older generation won’t have never touched drugs, alcohol, it was very ingrained in them that drugs or alcohol was forbidden, and they passed that onto their children. But I think now, the new generation are very open to dealing drugs, to taking drugs, taking alcohol. Although they won’t do it in front of their parents or friends openly, they might do it behind their backs, go to different cities, so their families aren’t aware of it, or their friends aren’t aware of it and do it in secret.”*

P8—family member of a person who used drugs

One participant stated that they felt the older generation chose not to acknowledge addiction within their family until it had escalated, with the limited intervention having a harmful impact on the younger generation within the family:

*“My grandparents didn’t really see it as a problem, until it was too late. And by that point, you’ve already damaged another generation and then it’s had an impact as you go on and I think that’s across the board.”*

P3—daughter of a person who used drugs

A concern raised by a community member about the lack of conversations had by the older generation regarding addiction was that the younger generation were not able to be educated by the older community about D&A use, and instead were learning about drugs and alcohol from their peers:

*“A lot of the parents will not approach the subject, will not talk about drink, will not talk about drugs. Will not… They just… They try to keep it all hush-hush, but the kids learn so much from school, when they’re in like, infants and juniors and like, are nine, ten years old, they learn so much. And culturally, we are very backwards because we don’t teach our kids anything. We don’t tell our kids that you mustn’t drink… You know, it’s bad for you and if you do or if someone tries to, you can say “no” because we culturally don’t have those conversations with our kids.”*

P9—family member of a person currently using drugs

### 3.2. Drug and Alcohol Use as a Taboo Topic and the Impact of Admitting Use

This theme incorporates data regarding families trying to hide use/not speaking about it and hiding it from the community due to perceived and/or enacted stigmatisation. The taboos maybe viewed as obstacles within the community to accessing drug and alcohol treatment when approaching recovery. Hence, admitting use has the potential to result in being ostracised from the community and impacting the reputation of the family. This leads to the perception of there being ‘no way out’—individuals feel that the only options available to them are prison, relocation within the UK or leaving the country.

The majority of participants interviewed reported that addiction is often kept hidden from the community by the family for fear of shame and being stigmatised:

*“No one talks about it. No one wants to talk about it. Everyone wants to put their head in the ground, and they don’t want to deal with it. Especially parents. I find they’re the ones… Because obviously for them, it’s a shameful thing. It’s an embarrassing thing for them. In the community–the community’s small. People find out.”*

P10—community leader

One participant highlighted that hiding aspects of a person’s life is not exclusive to just D&A use, and wider issues were at play. A concern raised around not disclosing addiction beyond the family is that it restricts the individual from being able to access treatment and seek recovery, with them being encouraged to engage in D&A use in secret:

*“Families tend to try and hide a lot of what’s going on and keep it kind of within the family. I think, when you’re doing that, you’re promoting this person not getting support, but also not owning that they’ve got a problem. And then when you start doing that, your kind of giving this person a comfort blanket to keep doing what they’re doing, keep making the mistakes they’re making, and it just keeps going round and round in circles.”*

P3—daughter of a person who used drugs

As seen above, there are several issues discussed; firstly, the desire for families to keep D&A use ‘unseen’ creates a space for the person using drugs to do so secretly without accessing any service intervention, creating a vicious cycle of drugs being consumed and concealed. Family members themselves feel trapped in this cycle, which they do not know how to escape; and family members may carry the shame and act as ‘buffers’ between the outside (potentially judgmental) community and the person with an addiction. Additionally, due to BSA and Muslim families feeling unable to access support through traditional treatment routes—they do not have the correct information to support family members and provide recovery support.

A participant with a family member currently using drugs reported that they felt unable to talk with friends and community members, stating that this was due to drug use being “a stigma”. This suggested a lack of support from family members within their own community as they navigated familial substance use. The participant further disclosed that they felt there was “no point” in having conversations with their peers about addiction, as it would not lead to support. They also reported harsh language being directed towards their family members for his mental health. Thus, D&A use escalates prior to individuals accessing D&A treatment:

*“I don’t even talk about his mental health condition, ‘cos it’s a stigma. People like, laugh at it and things and say, ‘look, he’s barking; he’s crazy’. It’s this whole attitude that he’s crazy. You know, there’s no sympathy. […] it’s better and easier to get cancer than it is to [have anything] like that, because no one’s going to be understanding.”*

P2—parent of a person currently using drugs

As seen above, the participant stated that they felt it would be easier if their family members were to have a physical illness, as they believed there would be more understanding and sympathy towards their family member than seen when using drugs and alcohol and struggling with mental health. This transfers to family members of those using drugs and alcohol experiencing shame when approaching D&A support:

*“I guess [there is] a stigma attached, like embarrassment, in terms of actually going to access those services, as you might with other healthcare services.”*

P7—community leader

In this instance, it is suggested that the culture around the community of treating D&A as a taboo creates obstacles for someone to be able to have open treatment and seek support without judgement from their peers.

It was highlighted by a BSA and Muslim community member that D&A use can lead to people being ostracised from religious and community activities:

*“They asked the leader in the mosque, and they said whose name it was, and they said no. ‘We can’t take this said person because, yes, the intention is good, and you are trying to help him and you’re trying to get him on the straight and narrow path and you’re trying to get him off the drugs and stuff […] this guy is not allowed to go on the Jamaat, because he will be corrupting the rest of the kids.’”*

P9—family member of a person currently using drugs

This suggests that addiction can lead to a feeling of isolation for the individual, especially where it is a topic often not discussed by the community that is known to be highly stigmatised against. This narrative highlights how community leaders also stigmatise PWUD.

Stigma is not only experienced by PWUD but also by the family, with it reported that stigmatising attitudes and behaviours regarding drug and alcohol use can lead to ostracising to the extent of determining a family’s quality of life and opportunities within the community:

*“It had an impact on kind of marriages for the younger generations, the kind of friends you were going to make.”*

P3—daughter of a person who used drugs

The impact stigma has on the whole family can create an extra pressure for the individual using drugs and alcohol, with them potentially feeling burdened by the responsibility of how they represent their family to the community. The actions of the family and/or the individual with addictions have far reaching consequences, inclusive of them being viewed as unsuitable friends/partners:

*“You would obviously see them in the street and as a British Muslim, you don’t want to see your family member on the street like that, ‘cos they’re representing your community and your family like, in a bad light.”*

P8—family member of a person who used drugs

This can create an escalated use of drugs and alcohol prior to individuals accessing treatment/support. If someone is seen accessing treatment, the impact of this could be it being discussed by their peers, with the culture of “gossip” in the community being an obstacle in this way:

*“Just sort of the stigma of bringing their family down. Like, in the Asian community, your family are like, very important and if someone does something bad, your reputation can get tarnished, so I think that’s why a lot of people don’t really talk about it or go to these places.”*

P8—family member of a person who used drugs

It was noted that a visible recovery might help to reduce stigma and provide a perspective that addiction can happen to anyone:

*“As a family, we’ve been quite open, but obviously because we’ve had to deal with struggles of substance use in prison and what have you […] prison and that kind of world has become more apparent within families and because it’s become more apparent, and also with the younger generation, a lot of them get involved in gangs and things like that, it’s now like, kind of, ‘well, you can’t judge me. I’m not the only one going through that’ and now everyone’s got a perspective of: actually, this can happen to anyone.”*

P3—Daughter of a person who used drugs

It was also highlighted that when someone openly, in the community, seeks treatment, it may help to influence those not in recovery to do the same. It was suggested that even when a person is in recovery, they are still stigmatised within the community, with derogatory labels assigned to them, thus highlighting the enduring nature of stigma:

*“There is still a stigma attached to someone in your family accessing the services because it means they’ve got a substance use problem. And then if they’ve got a substance use problem, you’re stigmatised as being like, a junkie, or whatever the label that comes with that.”*

P3—daughter of a person who used drugs

Confidentiality being upheld was a large concern raised by community members when considering traditional D&A treatment for their peers because D&A use was a taboo subject. This fear was also observed by a D&A practitioner:

*“I’ve spoken to a client about this in the past, briefly, because she was from a South Asian community, and she had a friend who wanted to come into the service, but they wanted to be anonymous because they didn’t want anyone to know at all that might then tell their family that they were coming in to services. So, I think stigma does play a part in it and maybe like, the community not understanding addiction.”*

P5—practitioner working in a drug and alcohol service

An uncertainty was expressed regarding what to expect from D&A services and it was highlighted that an extra level of reassurance was needed that anonymity would be upheld because of the stigma:

*“I don’t know, to be honest, the depths of the services and how they are offered. You would expect there would be a level of professionalism of course, otherwise they wouldn’t be working, but I think maybe even if you can reassure parents that it’s all confidential, in the same way that coming to the mosque, they know that’s going to stay there confidentially. You know, if we knew that it was, then maybe we could reassure them as well.”*

P7—community leader

As seen above, it was suggested that a connection made between the service and the mosque would help, as they are often the first point of call and would be able to confirm to the person seeking help that treatment is confidential and trustworthy.

Multiple participants reported that due to the nature of the community being close-knit, it means there is often a circulation of rumours, with this creating a barrier to recovery due to concerns that details regarding their D&A treatment would be shared across the community. These fears were directed towards the need for privacy policies in treatment services. This barrier was noted as particularly evident when there are concerns that someone in the community will see them accessing treatment and share this with their peers:

*“They think, if another Asian is there [in the service], they’re going to pass it on to everyone. D’you know? That’s that stigma; that’s the mentality, maybe. Thinking, ‘oh my gosh, she knows. She’s a Muslim or she’s a Bengali, or she’s a Pakistani… She’s going to pass that on to someone else’. But that trust building is the first one, more than anything else. Awareness and trust building is more than knowledge. You know? And once you build trust, then you can build on…”*

P1—carer of a person who used drugs

It was highlighted that a fear of confidentiality being breached, and concern about sharing drug and alcohol use, is not only exclusive to the BSA community, but it can also be a fearful thing to those seeking help in any community:

*“It can be difficult to speak to someone about how you feel and kind of the life choices that you’ve made and the drugs that you’ve taken, ‘cos nine out of ten times a lot of people aren’t completely honest with their families and that’s not just with Muslims; that’s across the board.”*

P3—daughter of a person who used drugs

### 3.3. Knowledge of Treatment Services 

This theme incorporates the extent to which the BSAs feel they are knowledgeable regarding addiction and services supporting those in recovery, as well as how education and/or correct information in this community can be further implemented to enhance accessibility.

A carer who had accessed a family support service reported that when it came to seeking services, it was initially to educate themselves about D&A use as they supported a person with addiction rather than for emotional and/or physical support for themselves. Family members of those using substances reported that prior to their family member becoming involved, they had very little knowledge about D&A use, with them feeling they had to become experts in order to support their family. When discussing D&A use, there was more of an acknowledgement of men using drugs rather than women; however, it was perceived that there was an increase in BSA women engaging in substance use:

*“I think males use a lot of drugs and alcohol more, because of the pressure of society and girls have started now to use it more. Especially in the Asian community, a lot more girls are drinking alcohol, compared to what it was and before they were ashamed, but actually, within their circles, it’s acceptable and now it’s like… ‘Well why aren’t you doing it?’ rather than, ‘how dare you do it’, so like, the questions have changed, but I think again, that’s generational. But again, I think it’s more men using it, because of the stress of the society.”*

P8—family member of a person who used drugs

It was highlighted that due to the community’s denial of D&A use, understanding what addiction meant was more important for the families and friends at the forefront. The stigma around addictions leads to limited awareness from caregivers where their family members use drugs and alcohol; hence, a lack of awareness of what addiction is was felt to be a barrier in itself:

*“No one wants to talk about it. Very few people want to deal with it. Those who acknowledge it don’t know what to do with it. So, you’ve got a huge lack of awareness with parents.”*

P10—community leader

Due to limited knowledge amongst the community, it makes it difficult for members to recognise and identify when a loved one is using drugs:

*“Mum and dad weren’t aware, because it wouldn’t have been something that they were exposed to, or that they had ready information to recognise almost.”*

P7—community leader

The community leader in the above quote stated that awareness and recognition appear to be a contributory factor to accessing D&A services. The community ambassadors said that when parents came to them it was often at a crisis point.

When discussing what drugs are used in the community, most participants agreed that cannabis is the most prevalent within the community, with a local street even being labelled as “weed street” by community members:

*“The hashish is everywhere. I mean, I can tell you that down the road, they are growing it in their bedrooms; I can tell you they are using it at the bottom of the street constantly. Sitting there and smoking it. You can buy it on every single corner here.”*

P6—daughter of a person who used drugs

As seen above, cannabis was perceived to be very accessible in the community. Cocaine was also reported to be used and supplied by community members. It is interesting to note here that on the one hand there is a lack of knowledge and complete denial reported about addiction, but on the other hand there is evidence to illustrate that there is knowledge of the most prevalent drugs that were being used. Drug use being observed was disclosed both by individual family members and community ambassadors. The quote below states that there had been a noticeable increase in the presence of cocaine. Of further interest was the fact that there was greater mention of drug use compared to alcohol, which was also the remit of the study:

*“We have one guy who comes down the street every day and brings little packets to some of the users further down the road and… The cocaine. The weed as well, but cocaine is quite… Getting quite stronger here now.”*

P6—daughter of a person who used drugs

It was reported by a community leader and family member that they were aware of county-lines cases within the community, in which children had been recruited as runners:

*“I know there’s children have been recruited to be like, runners, to go in between houses and distributing it.”*

P7—community leader

Steroid use was also highlighted to be a recent drug encountered, with it reported that young males in the community were using this. It was unclear whether these were being injected safely, and needle exchanges were being utilised:

*“What else has been a big one in recent years [is] steroid use and growth hormone. For the young lads, it’s rampant at the moment.”*

P10—community leader

Those interviewed who had accessed family drug and alcohol support reported a positive experience in which practitioners came to them in the community, adapting to where they felt comfortable such as beginning contact over the phone until they felt confident to meet in person at a place of their choosing. This was reported to be similar in traditional D&A treatment services, as supported by a parent of someone accessing services at the time. It is noted that there was flexibility and accommodating actions by the recovery service providers:

*“They were actually quite good; they came out; they really did try with him in this scenario, ‘cos they came out, they used to try and give him counselling sessions and talk to him. They even were willing to do home visits […] I must admit, they were really good with him. They really made an effort, ‘cos they normally don’t bother with the people who just do cannabis, but they came out, they gave him a couple of home visits and then the lady who was like, his case worker, she left and then a gentleman took over and even though he was only doing visits at the library, consultations, he would like, make an effort to try and come and see him at home”*

P2—parent of a person currently using drugs

Although services are able to adapt to the needs of the person accessing them, there were concerns raised around how well-known services are to the community, with this potentially being a barrier when approaching treatment services. It was suggested that a way to rectify services being unfamiliar to the community is for services to conduct outreach events in local community centres and mosques, as well as to make themselves known to community leaders and influential members of the community.

This need for outreach in the community was supported by multiple participants, with a family member stating that they felt being visible in the community as a service would encourage people to attend whilst also encouraging conversations between community leaders and services around how to improve accessibility:

*“They need to start going into places where they’re going to see a lot more of the community, so whether it’s, you turn up to events, go and speak to an Imam, tell the Imam that, ‘look, we’ve got this service, we want it accessible, you can give out leaflets…’ You need to start going into these communities and being a voice.”*

P3—daughter of a person who used drugs

When discussing if stigma was experienced by community leaders and imams, participants reported that they did not feel stigmatised, and instead found imams and leaders to be encouraging and supportive of recovery. A community leader reported that a barrier they faced when it came to providing support was that they felt unfamiliar with services, which affected how confident they felt in signposting someone using drugs and alcohol:

*“So we have a lot of people who come in from our community and they come in to drop in clinics or appointments at the mosque and they usually are parents of, maybe, teenagers or older children who have problems with addiction involving drugs and alcohol as well, and usually we talk to them and try and signpost them […] I wish there was more that we could do. I wish there was like, a more open service, or we had more information, to say, ‘go to this… Pick up the phone, phone this person, access this service, or go to this place…’ […] To be honest, I wouldn’t be comfortable referring people I know to those kind of places, because I don’t know if they would be accessible to them; I don’t know if they would be comfortable, just because lots of the people within the community that I work with, one of the things is that I always refer them to places either I’ve used myself or I’ve gone there and I know what it’s like […] It’s almost like you’ve got a sense of responsibility, ‘cos if you’re referring them to somewhere, you want them to feel comfortable and safe once they’re there and that they can belong, like you would do to any other group”*

P7—community leader

When discussing drug and alcohol treatment services and recovery groups, it became evident that those interviewed either had limited knowledge of services available or experienced a disconnect between services on offer and awareness of those services in the community. The majority of participants believed there to be no recovery support in the area, or if there was, for it to be expensive to access:

*“He’s been on a number of prescription tablets, but then because the facilities weren’t there, we’ve had to go private and try to get him counselling and get him into rehab, which is quite expensive.”*

P11—carer of a person currently using drugs

The above suggests that some members of the BSA community are unaware of the local open-access and free-of-charge D&A treatment services that are available. The limited knowledge left some interviewees reporting that they felt treatment had not been offered to them. The participant below said that her family member felt they had not been offered the appropriate support, with the family feeling they had lost faith in all services:

*“Nobody has ever, this has been going on for about 30 years, nobody has ever offered to help him in any way. Nobody offered to get him off drink; nobody’s offered to help get him off drugs. Nobody’s offered to help him get off all of the tablets [for mental health the GP] they give him.”*

P9—family member of a person currently using drugs

A family member of someone currently using drugs reported that they felt the only intervention appropriate for their family member was for them to go to prison, with this also demonstrating limited understanding of recovery and accessibility of D&A use:

*“This woman, the mum, was begging them: put him in hospital. He needs help. Can you get him help. Arrest him. Lock him up. She would prefer for him to be locked up in prison because he will get better. And that’s what this world, society has done. The drink, the drugs, because there is no help and support there.”*

P9—family member of a person currently using drugs

A family friend of someone currently using drugs and alcohol felt that the only option was to leave the city, with them believing rehabilitation was the only form of recovery, and that this was not accessible in this part of the United Kingdom. The participant stated that the GPs were unable to help them, although this was not further probed to understand what kind of help they were seeking from the GP practise and in what context support was requested:

*“There is no health centre here; the GP cannot help you. You are just all on your own and you are playing in the hands of drug dealers. […] At least he would have gone to xxx[city] or [city] or anywhere else, in a rehabilitation centre but I haven’t seen any rehabilitation centre here. Please correct me if I’m wrong. What is here? It’s nothing.”*

P4—family friend of a person currently using drugs

In order to combat D&A use, it was reported by a couple of participants that community leaders and family members often believe leaving the country to be one of the only options when it comes to recovery. It was noted that the family were still abroad at the time of interview, and it was unknown if the treatment was better or effective for them abroad:

*“After a few months, they decided to leave [city], to leave the UK for the health and life of her son. He was in danger. He was also married by then. Then she took this boy to Pakistan; they went over there… Obviously everyone had to sacrifice from here for six, seven months.”*

P4—family friend of a person currently using drugs

As mentioned above, this has a significant impact on the family, and it was reported by a parent that once their child had returned, they did not access relapse prevention services and continued to use drugs once back in the community. An issue raised regarding community members not accessing treatment is that where no professional intervention is happening, there is an added pressure on family members as the person using drugs and/or drinking alcohol is reliant on their family for recovery support. The pressure to provide recovery support to a family member where there is limited expertise is not only challenging for the person giving the support but also for the person using drugs who is not receiving psychosocial intervention but is simply being told that what they are doing is wrong:

*“At the moment, we’re just telling him that he can’t think that because he’s a Muslim anyway and it’s against our rules and against our religion, so we’re telling him off on that point.”*

P2—parent of a person currently using drugs

### 3.4. How to Increase Awareness and Accessibility of Treatment

This theme discusses how accessible treatment is perceived to be in terms of whether it is believed to uphold Islamic views and beliefs and whether services are seen as adapting to cultural needs.

Participants stated that there was a need for D&A services to be specifically tailored towards the BSA and Muslim community locally. They provided lengthy accounts of the importance of having diverse services that are open to all cultures so as not to ostracise a whole community.

A lack of awareness regarding D&A addiction was a crucial factor for the first-generation community. Recommendations to enhance current traditional D&A treatment services as well as the development of culturally appropriate recovery places for sustainability, were discussed. A family member reported that they felt treatment did not prioritise a person’s religion in the same way the individual does, with this having an impact on how applicable treatment can be to someone’s lifestyle:

*“I think if I was to access the drug and alcohol service, for me, the most important, regardless of how far down the road I’ve gone down, I’ve grown up Muslim. So first and foremost, religion is going to play a huge part in how I access the service and what it does for me. It’s the same as if I go and access a counsellor. Now, there’s a lot of information that people can give you or support you. But if they don’t have the same belief that you do, or values that you do, or they can’t help you in a lifestyle that you need help with, that works for you, it’s going to have a detrimental effect on how you move forward. So for example, the NA, AA, all these kinds of groups, even though they’re based on the 12-step programme and it is God-orientated, it’s Christian-orientated and don’t get me wrong, the bible, well, the Old Testament, and the Quran are quite similar, but the higher power and all those kinds of things, there’s got to be kind of a middle ground to meet the two and at the minute, I don’t think those services… And also, when you come into these kind of services and you’re talking about the guilt that you carry and all these kind of things… You’re talking about how sinful you feel and how guilty you feel for… Not just for what you kind of put your family through, but kind of between you and God.”*

P3—daughter of a person who used drugs

The above demonstrates how it is not a simple case of promoting treatment for everyone, but there also needs to be a knowledge of how Muslims and those in the BSA community view the world, their values and how they approach guilt from D&A use. It was remarked that this level of understanding is still applicable to those who are not practising Muslims anymore as, due to their upbringing, they may still identify with Islamic views. Further research is needed to explore the intersection of guilt, religious beliefs and substance abuse recovery, as seen in similar studies exploring spirituality as a recovery tool:

*“If they don’t know the nuances and the cultural and religious backgrounds and beliefs and practices–even though that young person might not be practicing Islam–but they still identify with Islam.”*

P10—community leader

A concern raised when discussing D&A treatment was the language barrier, particularly for those belonging to the older generation, and for those families that do not speak English as their first language. However, the majority of participants said that it would be useful for D&A services to employ people who spoke their first language:

*“That would be useful, if people spoke the language, ‘cos then you can speak to the parents as well, who might not understand, I think, especially for the younger generation, if they’ve got a child that’s maybe addicted to drugs or alcohol and they need to have the translation as why it’s important for them to get the treatment, ‘cos some of the older generation might think: oh, we’ll just lock the child up in the house and they’ll be fine, which is not true, so they’ll need that translation there to help them.”*

P8—family member of a person who used drugs

When discussing this with a practitioner, they confirmed that there is the provision of a translator for those whose first language is not English when accessing traditional D&A treatment:

*“We can arrange translators which is great if they come. I mean I think we don’t really experience a massive amount of issues with that, and also we can like, any kind of help sheets and stuff like that […] there’s a way of translating them all into different languages, so if, and I know people who are key working clients, who don’t speak very good English have done in the past, they’ve printed out the worksheet in English and then they’ve printed it out in another language as well and then like, they’ve sat with it in English and the client’s sat with it in the other language and they can go through it together, and I think that’s worked in the past. But they’re the main two, I think, that we kind of utilise.”*

P5—practitioner working in a drug and alcohol service

In current traditional D&A treatment services, a family member did report that they would recommend D&A treatment services and groups to the BSA and Muslim community, but believe that they would need to seek religious support separately, with the perception of Muslims being that God is a vital part of full recovery:

*“I would [recommend services]. Personally, I would […] I would also then say that obviously if it’s someone that’s Muslim and they believe in God or whatever, then they’ve got to find their own kind of… what’s the word? Their own sort of journey with God in amongst the support that they’re getting, because… Yeah, ‘cos it all ties in.”*

P3—daughter of a person who used drugs

It was further highlighted that they believe there is no provision currently for Muslims to engage in religious practises, with prayer rooms not currently being provided. When talking with a practitioner, however, they reported that they have identified this need and are currently trying to make provisions for this.

A concern raised regarding the accessibility of treatment mentioned was that staff within traditional D&A treatment services are not diverse; therefore, multiple participants reported feeling under-represented from the onset. Participants articulated that staff within traditional D&A treatment services should be more diverse, to encourage engagement, and that they should proactively recruit staff and volunteers who are representative of the communities they serve:

*“Hiring for your company then has an impact on who engages with the service, I think. Because I think, if, from the top, if it’s all filtered down, it filters down right down to the bottom, so me going into a service and saying, ‘there aren’t things in there that cater to me’, how is then someone going to then come and access that service that then they know is not catered for people of my colour to work there in the first place. Because how is it then going to improve for them when they’re engaging with the service?”*

P3—daughter of a person who used drugs

This was further supported by a practitioner:

*“We’re not a diverse workforce and I do think that makes an impact on the clients because I think it’s really important for people to see people who like, look like them, or who can relate with them. And you know, we’ve only got two non-white members of staff in the whole team and I don’t know if that’s demographically representative of the city as a whole, but I do think it’ll probably make an impact. […] I know that it’s come up with clients before, where they’ve said that they like working with someone who can relate to them.”*

P5—practitioner working in a drug and alcohol service

It was also reported by participants that they had only observed white people accessing traditional D&A treatment services, so felt under-represented in who treatment was for. They did, however, believe this was only a concern when approaching services, and not once in service:

*“I think it’s probably more like, engagement in the first place, rather than disengagement. I think once people are in and engaged, then that… Then they’ll probably stay engaged. But like, actually coming in in the first place… ‘Cos I don’t know if you would really feel comfortable coming into a service if like, there was nothing there that kind of related to you in it, or if you just didn’t know about it because you didn’t know the service existed because it’s not kind of promoted within the community.”*

P5—practitioner working in a drug and alcohol service

## 4. Discussion

This study is among the first known to understand what the barriers and facilitators are for the BSA and Muslim communities regarding addiction recovery. Key stakeholders took part in the study including BSA and Muslim members with familial substance use, carers and community leaders.

An improvement in cultural competency in traditional D&A treatment services may support access and sustainability for BSA and Muslim communities. Findings suggested D&A addiction was a stigmatised and hidden topic amongst the community. BSA communities were experiencing hidden burdens of D&A familial use and were ready to accept cultural and religiously tailored D&A services despite community stigma, trust and confidentiality barriers. An important finding was that there was a disconnection between services on offer and awareness of those services in the community. This is a finding that has far more relevance beyond the locality interviewed.

Participants in this study included male and female participants, including those with familial substance use, D&A treatment staff, community leaders and ambassadors. Interestingly, there were limited discussions on the cultural and religious standpoints of the use of drugs and alcohol, but instead barriers and facilitators reported by the participants on access to traditional D&A treatment services. Previous studies on alcohol consumption patterns in younger populations, such as the one by Martinotti et al. (2017), highlight the influence of social pressures and community norms in shaping drinking behaviours, which is also reflected in the increased substance use among younger generations in the BSA and Muslim communities [51]. These findings illustrated the acceptance and readiness of the BSA and Muslim community to access D&A services as opposed to being concerned about the D&A associated shame and stigma in the community [2], albeit, at the time of the study, it was also reported that it was known that addiction was prevalent amongst Muslims living in Muslim-minority communities.

It is argued that more educational work is required across imams (leaders in Mosques) to make meaningful headway in recovery for the BSA community, such as open communication [20,52]. A qualitative study in Malaysia, reportedly one of the first to implement and operate D&A recovery programmes in mosque settings (36 male participants), demonstrated that mosques are viable venues for the use of community assets to address community-orientated long-term recovery management [53].

Our findings support the studies in that there appears to be a strong shift in perceptions from participants with lived experiences of familial drug use from the stigma attached [30,38,40] to a mindset that PWUD require recovery support. It was clear, however, that stigma remains an issue within the BSA and Muslim community. Our findings reveal that the community is struggling with how to overcome and resist this stigma and community members are ready to move forward with educational and nudge strategies to suggest/direct people to access and attend D&A treatment services. It is argued that addiction is associated with stigma for BSA communities, both in regard to drugs and alcohol. One participant voiced that her family felt prison was the only option of recovery for their family member. The data collected did raise some stark differences in opinions. For example, where some participants were not speaking about the prevalence of addiction, others were. Another comment about the lack of drug addiction in home countries such as Pakistan was contradictory, where research has shown it was prevalent in this country [54,55,56].

It is argued that there is a readiness from the participants in the northern part of UK to seek treatment on behalf of their family and friends and to disclose to the community their family substance use. This trauma was discussed by some participants in this study, with more work suggested to understand family needs. Mauseth et al. (2016) state that education is a principal element in the community, with limited education being a similar finding in our research [34]. It was clear from the narratives, in particular from the daughter of a person who historically used drugs, that an individual’s role in society is not only to be responsible for themselves, but also to represent others. The weight of responsibility attached to that representational role can presumably be really hard to bear. Another participant felt community members were now not in a position to judge others, with this being an interesting comment due to the implication that the process of destigmatisation starts by normalising the situation, rather than normalising understanding and acceptance of diversity. Further work is suggested in this area. This study brings new knowledge to academia as there is a mention of the workforce, which was a concern, with staff not being reflective of the community in terms of race and religion, with this leaving community members feeling under-represented in treatment. However, it is important to note that after creating a BSA/Islamic-oriented service, it cannot be assumed that other obstacles in the community such as their fears of confidentiality will be immediately resolved, with an extended period of time being required to build-up trust.

There were several limitations to this study. Firstly, the religion of all the community participants was Islam, meaning that this study was not reflective of all members of the BSA community following other religions, such as Hindu and Sikhism. Despite efforts from the research team and employing snowballing methods, there were no people who used drugs and/or alcohol who consented to take part in this current research. It is argued that participant recruitment was a challenge for this project because of the nature of the topic. However, it was the positionality of the researcher (ZS, also PI) who was living as part of the BSA community that enabled conversations and led to the interviews on addiction, making this study possible. It was felt that creating a trusting relationship with credible community ambassadors and providing safe spaces led to the successful in-depth data collection. Due to the project time constraints, just over ten interviews were conducted; had there been more time for relationship building at the beginning of the research, more interviews would have been possible. It is recommended that at the beginning of research projects involving SA communities on addiction, additional time is used to build up rapport and trusting relationships with community groups prior to interviews.

Further research exploring a broader BSA population is suggested, in order to investigate how the barriers noted in this research are dictated by religion versus culture. There were also no participants who had lived experiences of D&A use, which is felt to be a weakness of this study and a recommendation for future research to include. However, it is suggested that people from within the community should conduct the interviews, especially those who are familiar with the community, for deeper, insightful data. Significantly, it was clear that support for the person who struggles with addictions is required, but also their immediate social circle. Addiction is still perceived as a taboo subject amongst most of the BSA community, but intergenerational differences were at play. Interventions that are culturally and religiously underpinned are suggested to consider sustainability of addiction recovery amongst the BSA and Muslim community [26]. Many younger generations were using drugs and alcohol, and their families were struggling to find recovery support. It was suggested that imams and community ambassadors may play a pivotal role in the messaging regarding recovery.

## 5. Conclusions

Overall, findings suggested that addiction was still perceived as a taboo topic amongst most of the BSA and Muslim community, leading to family members and those using drugs and/or alcohol to conceal their use for fear of being stigmatised and shamed by their peers. It was evident that this led to repercussions, such as BSA and Muslim community members not accessing treatment when needed nor having the knowledge of what support was available to them in their local area. It is recommended that in order to rectify the limited awareness amongst the BSA community, there is a need for collaboration and better communication channels with community leaders (imams) and outreach from recovery-based services and groups, with the view of educating and raising awareness. Implementing cultural and religious interventions may reduce barriers and address stigma to support the BSA and Muslim community in drug and alcohol services.

## Data Availability

Data Availability Statements are available in section “MDPI Research Data Policies” at https://www.mdpi.com/ethics (accessed on 31 August 2024).

## Appendix A

**Table A1:** Participant information.

Participant Number	Role	Ethnicity	Religion	Current/Historic Drug Use of Family/Friend/Experience of Supporting Members of the BSA Community
1	Carer	Bengali	Muslim	Historic
2	Parent	Pakistani	Muslim	Current
3	Daughter	Pakistani	Muslim	Historic
4	Family Friend	Pakistani	Muslim	Current
5	Practitioner	White	Christian	Experience of supporting members of the BSA community
6	Daughter	Pakistani	Muslim	Historic
7	Community Leader	Indian	Muslim	Experience of supporting members of the BSA community
8	Sibling/Cousin	Pakistani	Muslim	Historic
9	Family/Friend	Pakistani	Muslim	Current
10	Community Leader	Pakistani	Muslim	Experience of supporting members of the BSA community
11	Carer	Pakistani	Muslim	Current
